# Diagnosing Oligoarticular Juvenile Idiopathic Arthritis in a Two-Year-Old: A Case Report

**DOI:** 10.7759/cureus.90285

**Published:** 2025-08-17

**Authors:** Peter Richa, Matthew Thomas, Olivia Keller, Skye Karastury, Madhura Butala

**Affiliations:** 1 Medicine, Lake Erie College of Osteopathic Medicine, Bradenton, USA; 2 Pediatrics, Ascension St. Vincent's, Jacksonville, USA

**Keywords:** antinuclear antibody (ana), jia associated disease, juvenile idiopathic arthritis (jia), oligo-arthritis, oligoarticular arthritis, oligoarticular juvenile idiopathic arthritis, pediatric rheumatology

## Abstract

Juvenile idiopathic arthritis (JIA) is a rare heterogeneous group of autoimmune disorders presenting in children and is characterized by persistent joint inflammation without a known cause. Here, we present the clinical course of a two-year-old girl who developed acute right knee swelling without preceding trauma. Laboratory findings, joint aspiration, imaging, and clinical diagnosis initially suggested reactive arthritis; however, the persistence of symptoms and the emergence of a second affected joint led to the diagnosis of oligoarticular JIA. Laboratory analysis revealed a markedly elevated antinuclear antibody (ANA) titer with a homogeneous pattern, supporting the diagnosis. The patient was treated with intra-articular corticosteroid injections after a multidisciplinary evaluation. This case underscores the importance of considering JIA in young children with persistent monoarthritis, highlighting its evolving presentation, and initiating early rheumatologic involvement to guide diagnosis, manage complications such as uveitis, and tailor treatment strategies.

## Introduction

Joint pain is a frequent complaint in the pediatric population, often attributed to benign or self-limited causes such as trauma, transient synovitis, or growing pains [1]. However, juvenile idiopathic arthritis (JIA) remains an important diagnosis to consider when joint symptoms persist or are accompanied by other symptoms [2]. JIA is a rare condition characterized by inflammation with an unknown etiology, with an estimated annual incidence of 1.6 to 23 per 100,000 children [3]. Although no definitive diagnostic test currently exists for JIA, a combination of laboratory tests, imaging studies, and clinical evaluation helps rule out other causes of potential pathological causes of joint swelling [2]. Erythrocyte sedimentation rate, C-reactive protein, red blood cell count, and white blood cell count are a few of the many lab values that can help guide the diagnosis [4]. Thus, JIA is often considered a diagnosis of exclusion and is represented as persistent arthritis lasting at least six weeks in a child under 16 years of age [2].

JIA is further complicated as there are several subtypes, including oligoarticular, polyarticular, systemic, psoriatic, enthesitis-related, and undifferentiated forms, each with distinct clinical characteristics [5]. JIA can affect any joint, with each subtype defined by the number of joints involved, oligoarticular involving fewer than four joints, and polyarticular affecting more than five joints [5]. Systemic JIA is typically associated with fever, rash, lymphadenopathy, and hepatosplenomegaly, whereas these features are not usually present in the oligoarticular and polyarticular subtypes [5]. Additionally, specific biomarkers, such as rheumatoid factor (RF), human leukocyte antigen (HLA)-B27, and antinuclear antibody (ANA), can help further classify and characterize the various types of JIA [5].

A significant proportion of these patients test positive for ANA, which is associated with an increased risk of asymptomatic chronic anterior uveitis [5]. Early diagnosis and intervention are essential to prevent joint damage, growth disturbances, and ocular complications [2]. Although no universal treatment guidelines exist, the management of JIA is best approached through a multidisciplinary team, integrating both pharmacologic and nonpharmacologic strategies to optimize patient outcomes [5]. Non-steroidal anti-inflammatory drugs, followed by glucocorticoid injections for more effective pain relief, are initially employed to manage symptoms [5]. The severity and prognosis of JIA are variable and, in some cases, unforeseeable; however, current literature has indicated remission rates exceeding 50% in patients off medication in the systemic and oligoarticular subtypes [6].

Here, we present a two-year-old girl who initially presented with monoarticular arthritis thought to be reactive in nature but ultimately progressed to oligoarticular JIA. The case underscores the importance of considering JIA in any child with prolonged joint swelling, even in the absence of systemic symptoms. Additionally, it emphasizes the importance of correctly diagnosing the specific subtype of JIA to customize treatment approaches, assess the disease prognosis, identify any existing risk factors, and treat any likely comorbid conditions.

## Case presentation

A previously healthy two-year-old girl presented to the emergency department with her family with the acute onset of right knee swelling. The parents first reported noticing the swelling upon the patient waking up that morning, and the patient proceeded to walk with a limp. Additionally, the patient was unable to straighten her right knee when attempting to bear weight. The family denied any history of trauma, fever, rash, or systemic symptoms. Her mother reported a mild upper respiratory infection approximately three weeks before presentation, but her other past medical history was unremarkable, with appropriate achievement of all developmental milestones.

On physical examination, vitals were normal, and the patient did not appear to be in any acute distress and appeared active. During musculoskeletal examination, right knee swelling was noted along with deformity and effusion. No erythema, ecchymosis, or crepitus was recognized. Normal pulses were palpated throughout the right extremity, with no abnormalities identified throughout the right hip or ankle.

Initial laboratory workup revealed a markedly elevated erythrocyte sedimentation rate (ESR) of 101 mm/hr and an elevated C-reactive protein (CRP) of 1.3 mg/dL (Table 1). The platelet count was elevated at 702,000/μL (Table 2). A knee joint aspiration was conducted and sent for joint fluid analysis, which revealed a negative gram stain, total nucleated cell count of 4,280 µL, and a 155,955 µL red blood cell count (Table 3). These findings were thought to result from a rheumatic process, as imaging revealed synovial hypertrophy and inflammation. This helped rule out other potential causes of the laboratory values, such as tumors or complications related to injections.

**Table 1:** Erythrocyte Sedimentation Rate and C-Reactive Protein Lab Values

Results Name	Lab Values	Units	Reference Range
Erythrocyte sedimentation rate	101	mm/hour	3.0-13.0
C-reactive protein	1.3	mg/dL	0.5-1.0

**Table 2:** Complete Blood Count With Differential NA: not applicable.

Results Name	Lab Values	Units	Reference Range
White blood cell count	9.5	K/µL	6.00-17.50
Red blood cell count	4.77	mil/µL	3.50-5.00
Hemoglobin	10.1	g/dL	11.2-14.3
Hematocrit	31.4	%	34.0-40.0
Mean corpuscular volume (MCV)	65.8	fL	75.0-87.0
Mean corpuscular hemoglobin (MCH)	21.2	pg	23.0-31.0
Mean corpuscular hemoglobin concentration (MCHC)	32.2	g/dL	30.0-37.0
Red cell distribution width (RDW)	16.7	%	11.5-15.5
Platelets	702	K/µL	150-450
Mean platelet volume (MPV)	6.7	fL	7.0-11.0
Neutrophils, absolute	2.7	k/µL	1.50-8.00
Neutrophils %	28.9	%	15.0-35.0
Lymphocytes %	58.7	%	41.0-71.0
Monocytes %	8.1	%	3.0-13.0
Eosinophils %	2.9	%	0.0-7.0
Basophils %	1.4	%	≤2.0
Lymphocytes, absolute	5.6	K/µL	3.00-10.50
Monocytes, absolute	0.8	K/µL	0.20-1.20
Eosinophils, absolute	0.3	K/µL	0.00-0.60
Basophils, absolute	0.1	K/µL	0.00-0.10
Platelet sufficiency	Increased		NA/-
Anisocytosis	Few abnormal		None seen
Ovalocytes	Few abnormal		None seen
Variant lymphocytes	Few abnormal		None seen

**Table 3:** Fluid Cell Count of Right Knee Aspirate

Results Name	Lab Values	Units	Reference Range
Color, fluid	Red		Colorless, pale yellow
Clarity, fluid	Slight abnormal		Clear
Body fluid red blood cell count	155,955	/µL	4.2-6.1
Body fluid total nucleated cells	4,280	/µL	0-200

**Figure 1:**
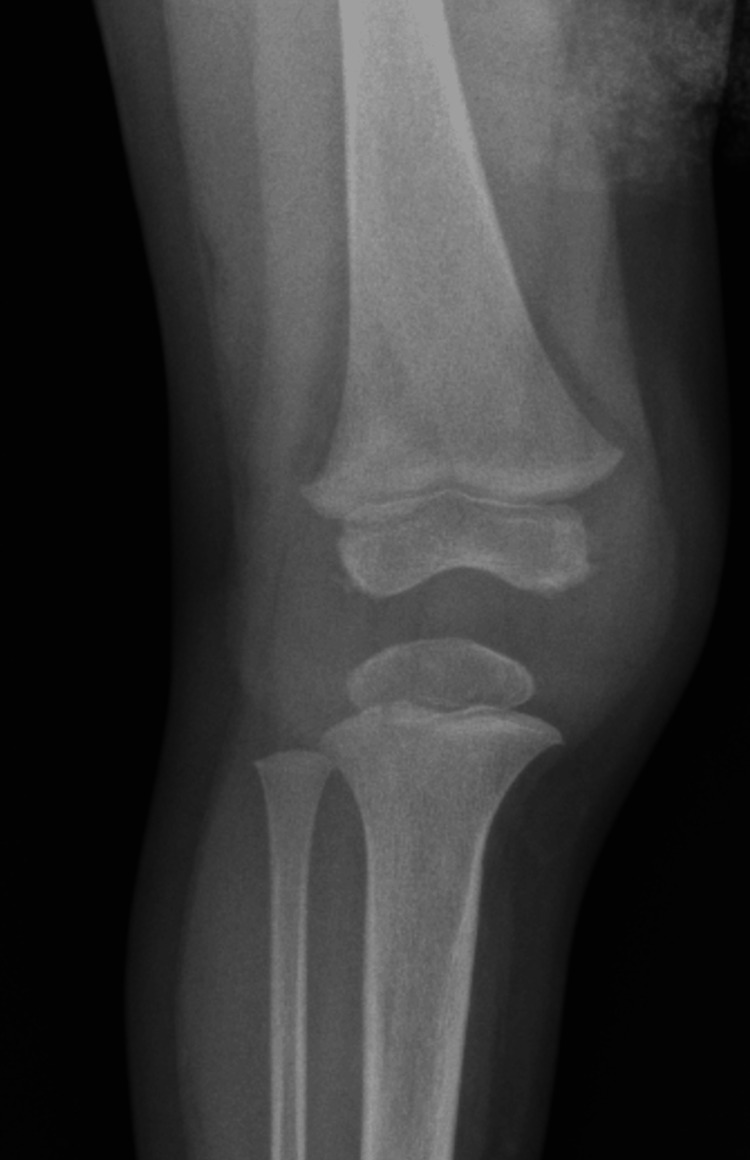
Anterior-Posterior Radiograph of the Right Knee Showing No Abnormalities

**Figure 2:**
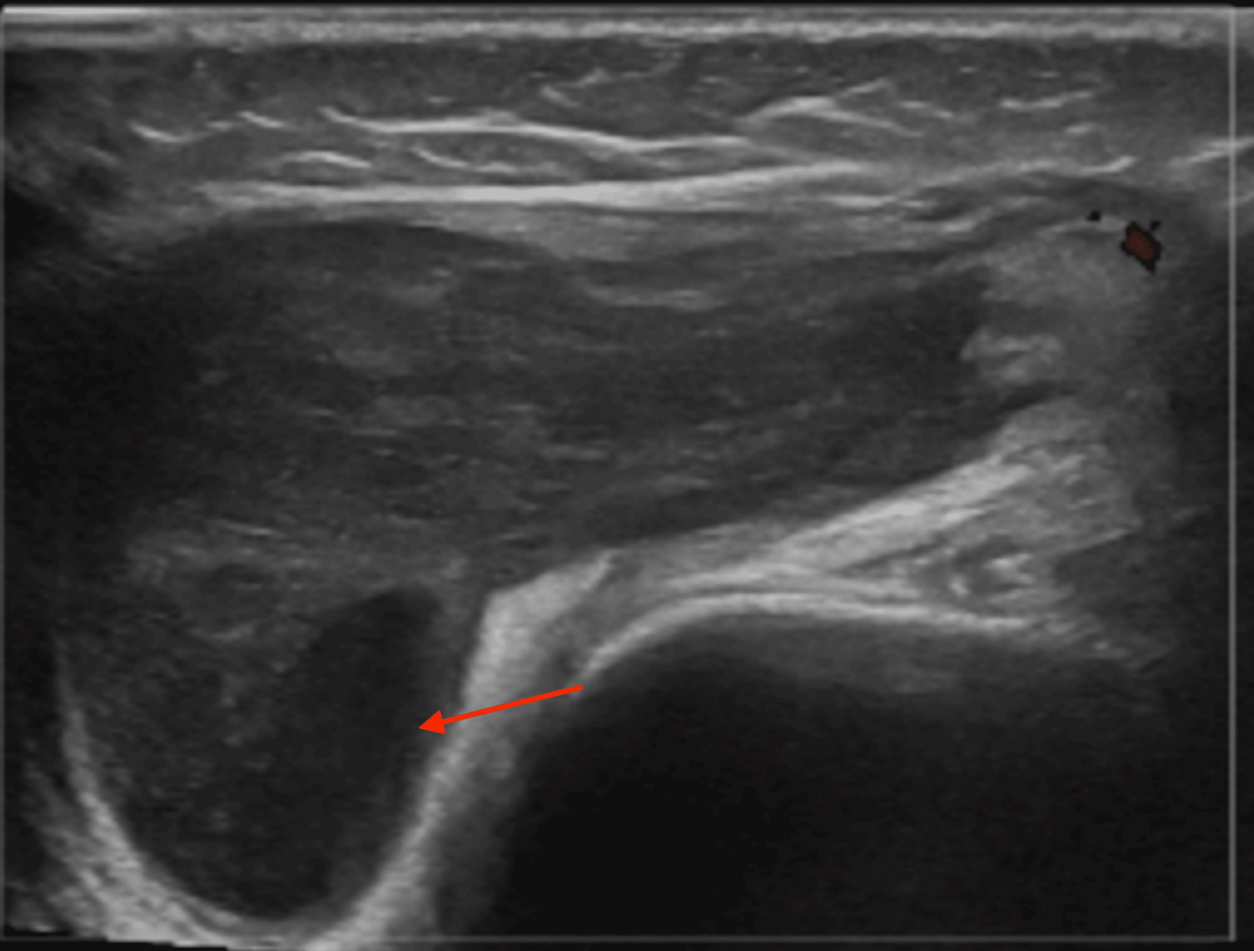
Ultrasound of the Right Knee Showing a Complex, Lobular Effusion Measuring 4.7x1.3x3.7 cm (Red Arrow)

**Figure 3:**
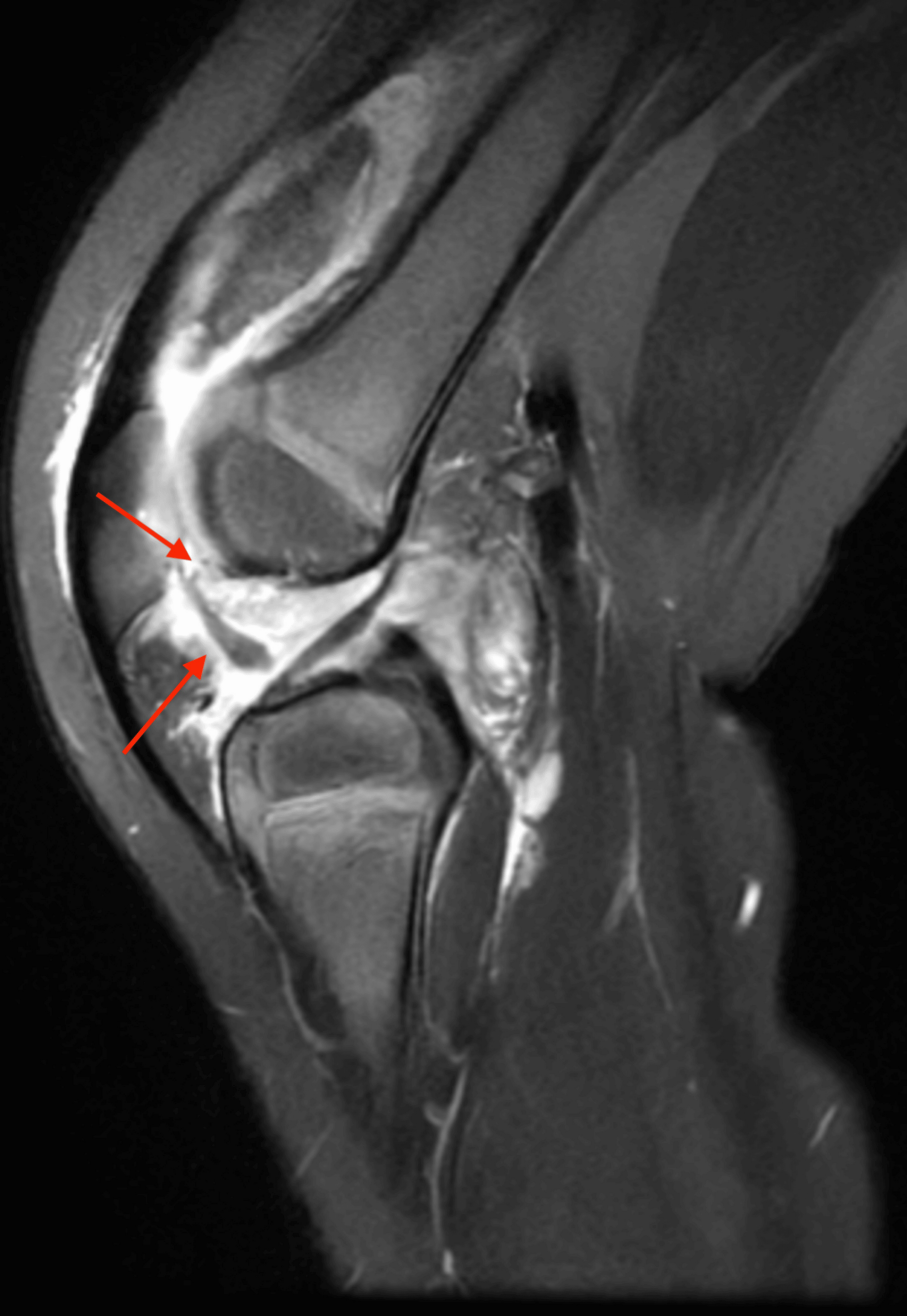
Lateral Magnetic Resonance Imaging of the Right Knee Showing Synovial Thickening and Irregularity Involving the Suprapatellar Recess, Patellofemoral Area, Infrapatellar Fat Pad, Anterior and Posterior Cruciate Ligaments, and Posterior Condyles (Red Arrows)

Diagnostic imaging of the right knee was conducted, including an X-ray, magnetic resonance imaging (MRI), and ultrasound. Radiographs of the knee were unremarkable (Figure 1). However, ultrasound revealed a complex, lobular effusion measuring 4.7x1.3x3.7 cm (Figure 2). This was further explored as the MRI demonstrated synovial thickening and irregularity involving the suprapatellar recess, patellofemoral area, infrapatellar fat pad, anterior and posterior cruciate ligaments, and posterior condyles (Figure 3). The joint was noted to be distended and contained non-enhancing material.

Based on these findings and the presence of recent viral illness, the patient was discharged and notified that her symptoms were most suspicious for reactive arthritis. Motrin, as needed, could be provided for the patient in the event she continues to experience discomfort at home. The patient’s family was informed that if symptoms persisted for more than six weeks, she would meet the criteria for JIA. The patient’s family agreed to be followed up by rheumatology at an outpatient clinic.

At a two-month rheumatology follow-up, the right knee remained persistently swollen, and the patient continued to walk with a limp. During the general examination, the patient remained active without showing any signs of distress. On physical examination, vital signs appeared normal; additionally, no fevers, rashes, or other joint issues were appreciated. There was swelling of the right second proximal interphalangeal joint. An extended musculoskeletal exam displayed mild atrophy of the right thigh and calf, with maintained muscle strength and sensation throughout the proximal and distal muscle groups bilaterally. The right knee was effused with warmth and tenderness to palpation. Importantly, the second proximal interphalangeal joint of the right foot was also effused, eliciting pain upon palpation.

Given the overall presentation and length of symptoms, the rheumatologist diagnosed the patient with oligoarticular JIA. The variability in radiologic studies and lab tests accompanying young patients with this diagnosis was explained. Additionally, the recommendation of an ophthalmologic exam due to the risk of uveitis, which increases in children less than six years old and those with a positive ANA, was discussed. The treatment options, including an intraarticular steroid injection, were suggested as they may be curative for some individuals with oligoarticular JIA. The family agreed to proceed with the joint injection for both the right knee and the right second proximal interphalangeal joint of the foot. ANA, RF, and HLA-B27 were also ordered to be assessed via aspiration at the time of injection.

About one month after the rheumatology clinic appointment, the patient underwent joint aspiration and injection performed by the interventional radiology team. Findings included a small effusion with extensive synovial thickening within the right knee. Nonetheless, a total of 20 mg of triamcinolone acetonide combined with 5 mL of 0.25% bupivacaine was administered via intra-articular injection into the right knee. This was followed by an injection of 8 mg of triamcinolone acetonide with 0.8 mL of 0.25% bupivacaine into the second proximal interphalangeal joint of the right foot. Post-operative analysis of the joint aspiration revealed negative RF, HLA-B27, and DNA antibody IgG (Table 4). Notably, however, ANA pattern 1 test revealed a homogenous pattern, with these findings being associated with systemic lupus erythematosus, drug-induced lupus, and juvenile idiopathic arthritis (Table 4). Additionally, ANA titer was 1:320, indicating a significantly elevated antibody level (reference range: <1:40 negative, 1:40-1:80 low, >1:80 elevated) (Table 4). After the procedure was successfully completed, the patient was discharged, and the family was advised to follow up with the pediatrician.

**Table 4:** Autoantibody Lab Values RNP: ribonucleoprotein, SSA/Ro: Sjögren's syndrome-related antigen A, ANA: antinuclear antibody, IFA: immunofluorescence assay, HLA: human leukocyte antigen, SSB/Ro: Sjögren’s syndrome-related antigen A, SSB/La: Sjögren’s syndrome-related antigen B, U1-RNP: U1 ribonucleoprotein, Jo1: histidyl-tRNA synthetase, SCL-70: DNA topoisomerase I, NA: not applicable.

Results Name	Lab Values	Units	Reference Range
Rheumatoid factor IgM	0.9	IU/mL	≤3.4
Rheumatoid factor IgA	1.4	IU/mL	≤13.9
DNA antibody IgG (double-stranded)	4.7	U/mL	≤14.9
Smith IgG	<0.7	U/mL	≤4.9
U1-RNP	1.2	U/mL	≤4.9
RNP-70Da	0.5	U/mL	≤6.9
SSA/Ro	0.6	U/mL	≤6.9
SSB/La	0.7	U/mL	≤6.9
Jo1 antibodies	<0.3	U/mL	≤6.9
Centromere antibody	0.6	U/mL	≤6.9
Anti SCL-70	<0.6	U/mL	≤6.9
ANA screen, IFA	Positive		Negative
ANA pattern 1	Nuclear homogenous		NA/-
ANA titer 1	6:20		<1:40 Negative, 1:40-1:80 Low antibody level, >1:80 Elevated antibody level
HLA-B27 antigen	Negative		Negative

## Discussion

As noted previously, the seven subtypes of JIA include systemic, oligoarticular, RF-negative polyarticular, RF-positive polyarticular, enthesitis-related arthritis, psoriatic arthritis, and undifferentiated JIA [7]. The International League of Associations for Rheumatology (ILAR) classification system provides the definition and criteria for each category [8]. This discussion will concentrate on the three primary subtypes, systemic, oligoarticular, and polyarticular, since the other subtypes typically have clinical features corresponding to one of these categories [7].

Currently, while not fully understood, the pathophysiology of JIA involves an autoimmune process in which the body’s immune cells, such as lymphocytes, macrophages, and neutrophils, attack its own cells [4]. Specifically, pro-inflammatory T-cells and autoantibodies initiate a cascade of cytokine release, driving joint inflammation through synovial infiltration [4]. This process results in synovial hyperplasia and hypertrophy, leading to pathological angiogenesis, swelling, and damage to the underlying cartilage and bone [4].

Emerging evidence highlights the pivotal role of genetic susceptibility in the pathogenesis of JIA [9]. Current literature indicates that siblings of affected patients are up to 30 times more likely than the general population to develop JIA [9]. The association of HLA class genes with the development of oligoarticular and polyarticular JIA suggests the effects of genetics [9]. On the other hand, there is also evidence suggesting the role of non-HLA genes in the development of JIA [10]. Nonetheless, these findings indicate the complex genetic relationship of genes and JIA. While not all-encompassing, testing for RF, ANA, and HLA may provide insight into the predisposition of JIA within certain family trees [10]. 

Additionally, environmental factors may influence the development of JIA; these may include infection, maternal smoking, and breastfeeding [10]. While no specific infectious agent or association has been identified, this is noteworthy as the patient in this case had recently experienced an upper respiratory infection [11]. Similarly, with controversial associations, the impact of cesarean section delivery and secondhand smoke exposure may predispose individuals [11]. The immunologic dysfunction and increased risk for infection associated with these two disorders cannot go unrecognized and, therefore, may have a role in the development of JIA [11].

In patients presenting with persistent joint swelling, several differential diagnoses are included in the workup, such as Lyme disease, arthritis associated with inflammatory bowel disease, malignancy, septic arthritis, transient arthritis, etc. [10]. In this case, the absence of systemic symptoms and normal gastrointestinal function made irritable bowel disease (IBD)-associated arthritis less likely [10]. The patient was afebrile and able to bear weight, reducing concern for septic arthritis [12]. Malignancy was ruled out through diagnostic imaging and joint aspiration, which showed no presence of malignant cells [10]. Additionally, workup labs including normal complete blood count (CBC), ESR/CRP, and joint fluid analysis supported the exclusion of infection and malignancy, while raising suspicion for juvenile idiopathic arthritis [12].

Due to the nature of JIA being a diagnosis of exclusion, making the correct diagnosis may prove to be a challenging task [2]. Diagnosing JIA requires a high index of suspicion, particularly in children under six who may not verbalize pain effectively. Laboratory findings such as elevated inflammatory markers and ANA positivity can support the diagnosis but are not definitive [13]. The ANA-staining pattern, characterized as homogenous or speckled, may provide further insight into the diagnosis [14]. ​​A homogeneous pattern, as seen in this patient's ANA analysis, is commonly associated with systemic lupus erythematosus (SLE) and juvenile idiopathic arthritis (JIA), whereas a speckled pattern is more typical of conditions such as Sjögren's syndrome and scleroderma [14]. Imaging can aid in detecting synovitis and joint effusions, particularly when physical examination is equivocal [2]. Oligoarticular JIA is often insidious in onset and can be misdiagnosed as transient synovitis or reactive arthritis, particularly when only one joint is involved [12]. In this case, the initial monoarticular presentation and recent upper respiratory infection led to a presumed diagnosis of reactive arthritis [15]. However, the persistence of symptoms and progression to a second joint underscored the importance of reassessing the diagnosis when clinical evolution does not follow the expected course.

Of note, there is a significant risk of progression or transition to the polyarticular subtype in patients diagnosed with the oligoarticular form [16]. This is most commonly observed within the first two years of the pathological course [16]. Considering these possible manifestations, adequate treatment and management of JIA are essential in promoting proper development and limited effects [2].

The long-term manifestations associated with JIA indicate the need for prompt diagnosis and treatment (Guillaume). Roughly 20%-25% of patients diagnosed with oligoarticular JIA will develop uveitis, identified as the inflammation of the anterior uveal tract and adjacent ciliary body [10]. Therefore, guidelines recommend frequent ophthalmologic screening in JIA patients, as uveitis can lead to vision loss if untreated [2]. Another long-term sequela that may be seen in JIA patients is musculoskeletal disturbances [2]. Temporomandibular joint arthritis has been found to occur in greater than 10% of patients with JIA, potentially leading to micrognathia if improperly treated [10]. Leg-length discrepancy, identified as asymmetric bony overgrowth in leg length and width, is another common complication of oligoarticular JIA [10]. Leg-length discrepancies greater than 1 cm may be associated with gait abnormalities [10].

While no strict existing guidelines exist, clinical treatment modalities depend on disease severity and joint involvement [5]. Intra-articular corticosteroid injections are first-line therapy for oligoarticular JIA and often result in prolonged remission [17]. Nonsteroidal anti-inflammatory drugs (NSAIDs) may also be used for symptom relief [2]. In refractory cases or those with extended joint involvement, disease-modifying antirheumatic drugs (DMARDs) such as methotrexate may be required [10]. These treatment interventions, while effectively managing JIA, are not without their own set of risks. For example, continuous and persistent use of corticosteroids is associated with an increased risk for infection, secondary to immunosuppression, and metabolic disturbances [6]. Ultimately, because the pediatric population is most affected by this disease, a multifaceted approach, incorporating pharmacological treatment, physical therapy, and psychosocial support-may be the most effective in mitigating its impact [6].

## Conclusions

This case highlights the evolving characteristics of JIA and the challenges involved in determining the potential causes of pediatric monoarthritis. Although JIA may present in a complex manner, ongoing patient follow-up and symptom monitoring can assist in refining the diagnosis. While the patient’s presentation initially aligned with reactive arthritis, eventual involvement of another joint and ANA reactivity provided clues to shift the working diagnosis to oligoarticular JIA. To ensure optimal outcomes, early referral to pediatric rheumatology, timely initiation of appropriate therapy, and consistent ophthalmologic screening are essential. This case reinforces the importance of considering JIA in any child with persistent or progressive joint symptoms.
